# Genetic polymorphisms in *MMP 2, 9 *and *3 *genes modify lung cancer risk and survival

**DOI:** 10.1186/1471-2407-12-121

**Published:** 2012-03-28

**Authors:** Patricia González-Arriaga, Teresa Pascual, Arturo García-Alvarez, Ana Fernández-Somoano, M Felicitas López-Cima, Adonina Tardón

**Affiliations:** 1 Departamento de Medicina, Unidad de Epidemiología Molecular del Cáncer del Instituto Universitario de Oncología del Principado de Asturias, Universidad de Oviedo, 33006 Oviedo, Spain; 2 Servicio de Neumología, Hospital de Cabueñes, Gijón, Spain; 3 CIBER Epidemiología y Salud Pública (CIBERESP), Spain

## Abstract

**Background:**

Matrix metalloproteases (MMPs) are proteolytic enzymes that contribute to all stages of tumour progression, including the later stages of invasion and metastasis. Genetic variants in the *MMP *genes may influence the biological function of these enzymes and change their role in carcinogenesis and progression. We have investigated the association between the -735 C/T, the -1171 5A/6A, and the -1562 C/T polymorphisms in the *MMP2, MMP3 *and *MMP9 *genes, respectively, and the risk and survival of lung cancer.

**Methods:**

The case-control study includes 879 lung cancer patients and 803 controls from a Caucasian population in Spain (CAPUA study). Genotypes were determined by PCR-RFLP. Odds ratios (OR) and 95% confidence intervals (CI) were calculated using unconditional logistic regression. The Kaplan-Meier method, long-rank test and Cox's were used for the survival analysis.

**Results:**

The *MMP9 *-1562 T/T genotype was associated with a statistically significant decreased risk of developing lung cancer (OR = 0.23; 95% CI: 0.06-0.85), whereas no association was found for the *MMP2 *-735 C/T and *MMP3 *-1171 5A/6A polymorphisms. The *MMP2 *-735 T/T genotype was statistically significantly associated with a decreased survival in non-small cell lung cancer (NSCLC) patients, identified as an independent prognosis factor of survival (hazard ratio (HR) = 1.79; 95% CI: 1.00-3.20). In contrast, no association was found between the *MMP3 *-1171 5A/6A and the *MMP9 *-1562 C/T polymorphisms and survival.

**Conclusions:**

These findings support the hypothesis that the *MMP9 *-1562 C/T polymorphism is associated with a protective effect against the development of lung cancer and suggest that the *MMP2 *-735 C/T polymorphism modify the length of survival in NSCLC patients.

## Background

Lung cancer is one of the leading causes of death worldwide. Approximately one million people, 850,000 men and 330,000 women [1], die from lung cancer per year. In Spain, lung cancer caused more than 20,000 deaths in 2008; of these, 17,135 were men, and 3,035 were women [2]. Despite some advances in the diagnosis and treatment of lung cancer in the last several decades, the prognosis of lung cancer remains poor. The 5-year overall survival rate of lung cancer is approximately 12% in Spain and < 9% in developing countries [3]. The discovery and application of specific prognostic biomarkers could improve the survival rate of lung cancer [4]. Although some efforts have been made in this field [5-9], stable biomarkers for both risk assessment and clinical outcome predictors of lung cancer are still scarce.

Matrix metalloproteinases (MMPs) are a family of proteolytic enzymes that are capable of degrading various components of the extracellular matrix. They are involved in all stages of cancer progression, not only in the process of tumour invasion and metastasis, but also in as proliferation, adhesion, migration, differentiation, angiogenesis, senescence, autophagy, apoptosis and evasion of the immune system [10]. The expression of these MMPs by tumour cells may help increase the invasive potential of tumour cells by allowing the remodelling of the extracellular matrix. In this sense, the overexpression of MMP2, MMP9 and MMP3 has been detected in various types of human cancer, such oesophageal cancer [11], gastric carcinoma [12], ovarian [13] and lung cancer [14,15], and has been significantly associated with tumour progression and decreased survival. Studies based on the generation of loss-of-function animal models have provided definitive evidence of the existence of MMPs with anti-tumour properties [16], which supports the idea of an emerging and paradoxical role of MMPs in tumour progression.

Functional polymorphisms in *MMPs *located in promoter regions may influence the expression of the proteins and thus contribute to individual differences in cancer susceptibility and prognosis. To date, a large number of studies have investigated the relationship between genetic variants in the *MMP2, 3 *and *9 *genes and lung cancer risk [17,18]. However, only few studies have explored the relationship between polymorphisms in such genes and lung cancer survival, and these studies have displayed conflicting results [19-21]. Three studies have been published that focus on non-small cell lung cancer (NSCLC). Rollin et al. showed that patients carrying the -735T allele in the *MMP2 *gene had a significantly longer survival time compared with those carrying the -735C/C genotype, whereas the -1562C/T polymorphism in the *MMP9 *gene was not associated with survival time [19]. Heist et al. demonstrated that the -735C/T and -1171 5A/6A polymorphisms in the *MMP2 *and *MMP3 *genes, respectively, did not modify the survival time in patients with stage I NSCLC [22]. Finally, Jin et al. showed that the -1562 C/T polymorphism in the *MMP9 *gene is significantly associated with survival [20]. Alternatively, there are no published studies that have analysed the association between polymorphisms in *MMPs *and survival time for small cell lung cancers (SCLC).

The main aim of this study was to investigate the relationship between 3 functional polymorphisms in the regulatory regions of the human gelatinases MMP2 and MMP9 and the human stromelysin MMP3 and lung cancer risk in the individuals from the CAPUA study. The study also investigated whether polymorphisms in the *MMP2, MMP3 *and *MMP9 *genes may modify the survival time among NSCLC and SCLC patients.

## Methods

### Study subjects

The detailed methods of recruiting participants for this hospital-based case-control study have been described elsewhere [8,9,23]. Briefly, case incidences of histologically confirmed lung cancer were recruited in two main hospitals of Asturias in Northern Spain [Cabueñes Hospital in Gijón and San Agustin Hospital in Avilés], which followed an identical protocol from October 2000 to June 2010 (CAPUA study). The controls were selected from patients admitted to participating hospitals for a list of diagnoses believed to be unrelated to the exposures of interest (See additional file 1: List of pathologies accepted for controls). The controls were individually matched to the cases on the basis of ethnicity, gender, and age (± 5 years). The main specific pathologies of the final controls selected were as follows: 41.1% inguinal and abdominal hernias (ICD-9: 550-553), 32.5% injuries (ICD-9: 800-848, 860-869, 880-897), 8.8% appendicitis (ICD-9: 540), and 13.3% intestinal obstructions (ICD-9: 560, 569, 574). The study was approved by the ethical committee of the hospitals, and written consent was obtained from each participant. A total of 879 cases and 803 controls agreed to participate in the study and were interviewed. Of these, 841 cases (95.7%) and 742 controls (92.4%) provided a blood or buccal cell sample. Until February 2010, when the DNA extraction and genotyping was completed, we had samples from a total of 841 cases and 657 controls. Sixteen individuals (13 cases and 3 controls) were excluded because of problems in the DNA extraction. The following individuals were excluded because of difficulties in genotyping, mainly because of poor quality DNA: 56 individuals (12 cases and 44 controls) were excluded for *MMP2*, 232 individuals (112 cases/120 controls) were excluded for *MMP3*, and 71 individuals (66 cases/5 controls) were excluded for *MMP9*. Thus, the final study population available for the analyses was 816 s/610 controls for *MMP2*, 716 cases/534 controls for *MMP3*, and 762 cases/649 controls for *MMP9*.

### Data collection

Information on known or potential risk factors for lung cancer was collected personally through computer-assisted questionnaires by trained interviewers during the first hospital admission for diagnosis. Structured questionnaires collected from each participant information on age, gender, sociodemographic characteristics, diet (including alcohol consumption), recent and prior tobacco use, and personal and family history of cancer (first-degree relatives). All eligible cases and controls included in our analysis were Caucasian.

Participants were categorised by tobacco consumption into three groups: those who have never been smokers, defined as subjects who had not smoked at least one cigarette per day regularly for six months or longer in their lifetimes; former smokers, defined as regular smokers who had stopped smoking at least one year before the interview; and current smokers defined as subjects who met none of the previous criteria. Smoking intensity (pack-years (PY)) was defined as the number of packs of cigarettes smoked per day multiplied by the number of years of smoking.

The dietary section of the questionnaire ascertained the frequency of consumption and usual portion size of 117 food items (including alcoholic beverages) and was used to estimate daily intake of alcohol and calories.

For each job held for a minimum of 6 months or longer, we obtained information on the industry name, production type, job title, and the year in which the job began and ended. Occupations and industries were coded using the 1977 Standard Occupational Classification (Office of Federal Statistical Policy and Standards, 1977) and 1972 Standard Industrial Classification schemes (Office of Federal Statistical Policy and Standards, 1972). Lastly, each coded occupation was categorised regarding whether it is included in list A.

### Genotype determination

The polymorphisms in the promoters of the *MMP *genes analysed in this study are shown in Table 1. The polymerase chain reaction (PCR) combined with the restriction fragment length polymorphism (RFLP) was used to determine the *MMPs *genotypes. Genomic DNA used for the assay was extracted from peripheral blood samples (96.5% of total samples) or exfoliated buccal cells (3.5% of total samples) as previously described [24]. For quality control, genotyping was repeated randomly in at least 5% of the samples, and two of the authors independently reviewed all results. Another quality control method was to take 50 blood and mouthwash samples from the same participants to ensure the reliability of the genotyping results of the mouthwash samples. In both quality controls, no differences were found. PCR reactions were carried out in a total volume 10 μl containing 20 ng of genomic DNA, 0.25 mM of each dNTP (Ecogen, Biologia Molecular S.L.), 0.2 units of Taq polymerase (Biotools, Inc.) and 2.5 pmol of each primer in a 1×PCR buffer (Sigma-Aldrich Co.). The details of the primers and PCR conditions used for the amplification of *MMPs *are shown in Table 1. A 5 μl aliquot of PCR product was digested overnight at 37°C with 0.4 units of the indicated restriction enzyme. After overnight digestion, the products were separated on agarose gels and stained with ethidium bromide (restriction enzymes are shown in Table 1). To verify that the data obtained by RFLP coincided with the allele sequence, representative fragments were sequenced (data not shown).

**Table 1:** Details of PCR and RFLPs conditions for polymorphisms studied

Gene	Polymorphism	Primer sequence	PCR Conditions	Enzyme
*MMP9*	-1562C/T	(F) GCCCGCTCTGGATTATACG (R) CTATCATCTCCTGGCCCCC	28 cycles: 94°C 30s, 65°C 30s, 72°C 30s	*Sph*I

*MMP2*	-735C/T	(F) ATAGGGTAAACCTCCCCACATT (R) GGTAAAATGAGGCTGAGACCTG	30 cycles: 94°C 30s, 62°C 30s, 72°C 30s	*Hinf*I

*MMP3*	-1171 5A/6A	(F) TTTCAATCAGGACAAGACgaaGTTT* (R) GATTACAGACATGGGTCACA	30 cycles: 94°C 300s, 53°C 30s, 72°C 30s	*Xmn*I

* modifíed bases

### Survival analysis

Survival questionnaires were collected by a pneumologist trained to treat lung cancer patients who had been diagnosed at least 24 months earlier. Thus, a total of 879 eligible cases were selected up until June of 2010. We evaluated the overall survival sub-divided by NSCLC (Non-Small Cell Lung Cancer) and SCLC (Small Cell Lung Cancer) and on the basis of their different histopathological presentation and clinical stages (NSCLC: I, II, III, IV; SCLC: extended (ES) or limited (LS)).

### Statistical analysis

Tests for the Hardy-Weinberg equilibrium among the controls were conducted using observed genotype frequencies and a χ^2 ^test with one degree of freedom. A univariate analysis was first performed to compare the distribution of age and gender and the frequencies of alleles and genotypes. The differences in the distribution between the cases and the controls were tested using the χ^2^, Fisher exact and the Mann-Whitney U-test, where appropriate. The crude odd ratios (ORs) were calculated by Wolf's method [25]. A multivariate unconditional logistic regression analysis was performed to calculate adjusted ORs and 95% confidence intervals (CIs), adjusting for age, gender, family history of cancer (first-degree relatives), tobacco consumption in pack-years, alcohol consumption, calories, and occupation. Survival time was calculated from the dates of lung cancer diagnosis to the date of death, which was collected from the databases of the National Death Index of Ministry for Health and Social Policy. The survival curves were constructed using the Kaplan-Meier method, and the differences between the groups were tested by the log-rank method. The multivariate analysis of the probable prognostic factors for survival was performed using Cox's proportional hazard regression analysis. The relative risk with 95% confidence intervals was assessed, adjusting for variables that were statistically significant in univariate analysis. All statistical analyses were performed with STATA version 8 software.

## Results

### Population characteristics

The study population consisted of 879 lung cancer cases and 803 controls drawn from the Caucasian population of Asturias, Northern Spain. The distribution of demographic characteristics and clinical data is summarised in Table 2. There were more current smokers (51.5% vs. 27.5%) and more heavy smokers (62.2 vs. 36.3 PY) among the cases than among the controls (*P *< 0.001). Histologically, NSCLC and SCLC represented the 81.9% and 17.1% of lung cancer cases, respectively. Regarding the clinical stage, 66.0% of NSCLC patients were in stage III-IV, and 34.0% were in stage I-II, whereas 47.7% of SCLC patients presented extended stage. The control population was consistent with the Hardy-Weinberg Equilibrium (HWE) for the polymorphisms in *MMP9 *and *MMP3*, but not for the polymorphism in *MMP2*. Given that the first standard source for deviation from the HWE is a genotyping error, the results obtained for this polymorphism must be interpreted with caution.

**Table 2:** Characteristics of lung cancer cases and controls patients of CAPUA study

Variable	Cases (n = 879) n (%)	Controls (n = 803) n (%)	*P*^a^
**Gender**			
Male	785 (89.3)	688 (85.7)	
Female	94 (10.7)	115 (14.3)	0.024
**Age **(yrs), mean (SD)	66.1 (10.6)	64.4 (11.2)	0.002
**Smoking Status**			
Never	53 (6.1)	231 (29.1)	
Ever	821 (93.9)	563 (70.9)	0.000
Former	369 (42.3)	337 (43.0)	
Current	449 (51.5)	215 (27.5)	0.000
**Pack-years**^b^, mean (SD)	62.2 (36.2)	36.3 (30.8)	0.000
**Family history of cancer**			
No	486 (58.3)	473 (60.3)	
Yes	347 (41.7)	311 (39.7)	0.416
Lung cancer	91 (10.9)	52 (6.6)	
Other cancers	256 (30.7)	259 (33.0)	0.009
**Histological types**			
Squamous cell carcinoma	358 (40.7)		
Adenocarcinoma	264 (30.0)		
Small cell carcinoma	150 (17.1)		
Non-differentiated	54 (6.1)		
Large cell carcinoma	24 (2.7)		
Others	15 (1.7)		
Clinical diagnosis	6 (0.7)		
Missing	8 (0.9)		
**Clinical stages** NSCLC			
I	186 (25.9)		
II	58 (8.1)		
III	245 (34.1)		
IV	229 (31.9)		
SCLC			
LS (Limited stage)	78 (52.3)		
ES (Extend stage)	71 (47.7)		
**Calories**, mean (SD)	2405.6 (845.4)	2276.9 (700.4)	0.045
**Alcohol consumption **(gr), mean (SD)	26.4 (39.7)	22.2 (35.5)	0.033
**Occupation (list A)**			
No	703 (80.7)	677 (87.1)	
Yes	168 (19.3)	100 (12.9)	0.000

^a ^Two-sided χ^2 ^test and Mann-Whitney where appropriate

^b ^Pack-years for ever smokers

The characteristics and clinical data of NSCLC and SCLC patients are shown in Table 3. To date, 666 NSCLC (92.4%) and 137 SCLC (91.3%) cases have died. Regarding the NSCLC patients, the clinical stage and surgery status were associated with survival time (long-rank p < 0.000). Furthermore, the univariate Cox regression analysis showed that the risk of death for NSCLC was significantly associated with all clinical stages, and late diagnosis is the clinical characteristic most associated to death (compared with stage I, adjusted HR = 1.45; 95% CI: 1.05-2.00 for stage II; adjusted HR = 2.26; 95% CI: 1.83-2.79, for stage III; and adjusted HR = 3.97; 95% CI: 3.19-4.94 for stage IV), whereas the surgical operation decreased the risk of death (adjusted HR = 0.32; 95% CI: 0.27-0.39). Alternatively, when the SCLC patients were analysed, we found a significant increased risk of death between ever smoking (adjusted HR = 2.51; 95% CI: 1.09-5.75) and individuals with extended stage (adjusted HR = 2.19; 95% CI: 1.54-3.11), whereas patients who received chemotherapy and radiotherapy showed a significant decreased risk of death (adjusted HR = 0.41; 95% CI: 0.26-0.62 and HR = 0.46; 95% CI: 0.32-0.64, respectively).

**Table 3:** Characteristics and clinical data of 721 NSCLC and 150 SCLC patients of CAPUA study

Variables	NSCLC							SCLC						
	
	Patients		Deaths	MST (months)	Log-rank	HR	95% CI	Patients		Deaths	MST (months)	Log-rank	HR	95% CI
	n	%						n	%					
**Gender**					0.293							0.053		
Male	643	89.2	598	9.3		1.00		134	89.3	123	8.3		1.00	
Female	78	10.8	68	12.5		0.87	0.68-1.12	16	10.7	14	14.5		0.57	0.32-1.02
**Age (years)**					0.083							0.041		
≤ 68	375	52.0	342	11.3		1.00		79	52.7	69	9.8		1.00	
> 68	346	48.0	324	8.2		1.15	0.98-1.34	71	47.3	68	7.6		1.42	1.01-2.00
**Smoking Status**					0.585							0.025		
Never	45	6.3	41	10.5		1.00		7	4.7	6	54.2		1.00	
Ever	672	93.7	621	9.5		0.92	0.67-1.26	142	95.3	130	8.4		2.51	1.09-5.75
Former	307	45.8	284	9.4	0.809	0.90	0.65-1.25	59	41.8	54	7.5	0.022	2.88	1.29-6.43
Current	363	54.2	335	9.5		0.93	0.67-1.28	82	58.2	75	9.2		2.24	1.02-4.91
**Histological Types**					0.608									
Squamous cell carcinoma	358	49.6	335	10.9		1.00								
Adenocarcinoma	264	36.6	240	10.2		1.05	0.89-1.24							
Others	99	13.7	91	7.8		1.12	0.89-1.43							
**Clinical stages**					0.000									
I	186	26.1	151	26.1		1.00						0.000		
II	58	8.1	51	19.7		1.45	1.05-2.00							
III	244	34.3	238	9.6		2.26	1.83-2.79							
IV	224	31.5	220	5.2		3.97	3.19-4.94							
Limited stage (LS)								78	52.3	69	12.2		1.00	
Extend stage (ES)								71	47.7	67	5.8		2.19	1.54-3.11
**Surgical Operation**					0.000									
None	523	72.5	509	7.2		1.00								
Yes	198	27.5	157	30.9		0.32	0.27-0.39							
**Chemo or Radiotherapy**					0.469									
None	301	41.7	262	6.9		1.00								
Yes	420	58.3	412	11.7		1.06	0.90-1.24							
**Chemotherapy**												0.000		
None								29	19.3	28	1.6		1.00	
Yes								121	80.7	109	9.9		0.41	0.26-0.62
**Radiotherapy**												0.000		
None								74	49.3	71	5.4		1.00	
Yes								76	50.7	66	12.1		0.46	0.32-0.64

NSCLC: non-small cell lung cancer; SCLC: small cell lung cancer; MST: median survival

### Associations between the *MMP *genotypes and lung cancer risk

We examined the association between polymorphisms in the *MMP2, 3 *and *9 *genes and lung cancer risk (Table 4). A total of 816 cases and 649 controls were genotyped up until February 2010 (816 cases and 610 controls for the polymorphism in the *MMP2 *gene, 716 cases and 534 controls for the polymorphism in the *MMP3 *gene, and 762 cases and 649 controls for the polymorphism in the *MMP9 *gene). The variant genotype -1562 T/T in the *MMP9 *gene was associated with a decreased risk of developing lung cancer (adjusted OR = 0.23; 95% CI: 0.06-0.85; *P *= 0.027). When we carried out the stratified analysis by selected variables, an association was found between the polymorphism in the *MMP9 *gene and the lung cancer risk for age and smoking status (adjusted OR = 0.07; 95% CI: 0.01-0.58; and adjusted OR = 0.28; 95% CI: 0.08-1.01, respectively) [Data not shown]. In contrast, no association was found between the -735 C/T and -1171 5A/6A polymorphisms in the *MMP2 *and *MMP3 *promoter genes and lung cancer risk (adjusted OR = 0.86; 95% CI: 0.35-2.13; *P *= 0.749 and adjusted OR = 1.19; 95% CI: 0.84-1.67; *P *= 0.331, respectively).

**Table 4:** Analysis of polymorphisms and lung cancer risk in CAPUA study population

*MMPs*	Genotype	Cases n (%)	Controls n (%)	**OR**^a^	[95% CI]	*P*	*P *trend
***MMP9***	C/C	581 (76.2)	483 (74.4)	1.00			
	C/T	174 (22.8)	148 (22.8)	0.96	0.69-1.35	0.830	
	T/T	7 (0.9)	18 (2.8)	0.23	0.06-0.85	0.027	0.202

***MMP2***	C/C	596 (73.0)	465 (76.2)	1.00			
	C/T	206 (25.2)	125 (20.5)	1.18	0.83-1.67	0.359	
	T/T	14 (1.7)	20 (3.3)	0.86	0.35-2.13	0.749	0.601

***MMP3***	6A/6A	185 (25.8)	139 (26.0)	1.00			
	6A/5A	367 (51.3)	276 (51.7)	1.19	0.83-1.72	0.339	
	5A/5A	164 (22.9)	119 (22.3)	1.17	0.76-1.81	0.483	0.331

^a^Adjusted by age, gender, pack-years, family history of cancer, occupation (list A), alcohol consumption (gr), and calories

### Associations between the *MMP *genotypes and NSCLC and SCLC survival

**Figure 1:**
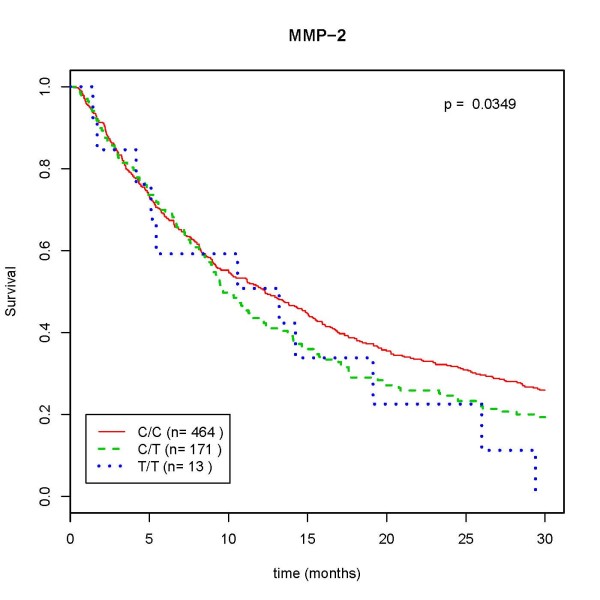
**Kaplan-Meier survival curves of patients with NSCLC by MMP2 genotypes, CAPUA study population, 2001-2010**. The individuals with T/T genotype showed significantly lower survival rates than the individuals with the C/C genotype.

When the Kaplan-Meier survival curves and the Cox regression analysis were used to assess the associations between the *MMPs *polymorphisms and survival time, NSCLC cases with the *MMP2 *-735 T/T genotype showed a lower survival time than the individuals with C/T or C/C genotypes (*P *= 0.035) (Figure 1). In addition, the multivariate analysis used to delineate significant prognostic factors for survival, showed that the T/T genotype in *MMP2 *was an independent prognostic factor for overall survival after adjustment for age, gender, pack-years, histological types, clinical stage, surgical operation status and chemotherapy (adjusted HR = 1.79; 95% CI: 1.00-3.20) (Table 5). However, the patients with SCLC and the -735 T/T genotype did not have statistically significant results (adjusted HR = 1.25; 95% CI: 0.17-9.31) (Table 6). However, individuals with the *MMP3 *-1171 5A/5A or the *MMP9 *-1562T/T genotypes did not show a statistically significant association with survival time either in NSCLC patients (adjusted HR = 0.92; 95% CI: 0.72-1.18 and adjusted HR = 0.85; 95% CI: 0.32-2.30, respectively) (Table 5) or in SCLC patients (adjusted HR = 0.67; 95% CI: 0.35-1.27 and adjusted HR = 0.97; 95% CI: 0.29-3.25, respectively) (Table 6).

**Table 5:** Association between genotypes of *MMP9, 2 *and *3 *and NSCLC patients' survival of CAPUA study

Genotypes	Patients	Deaths	MST (months)	Crude HR	95% CI	HR^a ^	95% CI
***MMP9***	n = 625						
**C/C**	477	447	10.1	1.00		1.00	
**C/T**	144	131	9.3	0.91	0.74-1.10	1.03	0.84-1.26
**T/T**	4	4	12.8	1.17	0.44-3.13	0.85	0.32-2.30
***MMP2 ***	n = 668						
**C/C**	481	446	10.0	1.00		1.00	
**C/T**	174	164	9.4	1.10	0.92-1.32	1.12	0.93-1.35
**T/T**	13	12	10.6	1.37	0.77-2.44	1.79	1.00-3.20
***MMP3 ***	n = 593						
**6A/6A**	148	137	14.1	1.00		1.00	
**6A/5A**	300	282	9.1	1.16	0.95-1.43	0.95	0.77-1.18
**5A/5A**	145	135	9.3	1.09	0.86-1.39	0.92	0.72-1.18

^a^Adjusted by age, gender, pack-years, histological type, clinical stage, surgical operation and radio and chemotherapy

MST: median survival time

**Table 6:** Association between genotypes of *MMP9, 2 *and *3 *and SCLC patients' survival of CAPUA study

Genotypes	Patients	Deaths	MST (months)	Crude HR	95% CI	HR^a ^	95% CI
***MMP9***	n = 129						
**C/C**	98	88	9.5	1.00		1.00	
**C/T**	28	25	8.9	1.08	0.69-1.69	1.06	0.67-1.68
**T/T**	3	3	24.5	0.67	0.21-2.12	0.97	0.29-3.25
***MMP2 ***	n = 140						
**C/C**	112	102	8.8	1.00		1.00	
**C/T**	27	24	8.9	0.90	0.58-1.41	0.98	0.60-1.58
**T/T**	1	1	9.2	1.34	0.19-9.70	1.25	0.17-9.31
***MMP3 ***	n = 116						
**6A/6A**	37	34	7.6	1.00		1.00	
**6A/5A**	61	54	9.4	0.84	0.55-1.30	0.79	0.50-1.26
**5A/5A**	18	16	13.4	0.69	0.37-1.26	0.67	0.35-1.27

^a^Adjusted by age, gender, pack-years, histological type, clinical stage, surgical operation and radio and chemotherapy

MST: median survival time

## Discussion

We evaluated the effect of three polymorphisms in the promoter regions of two human gelatinases, MMP2 and MMP9, and the human stromelysin MMP3 on the risk and survival time of lung cancer.

In this study, the distribution of the MMP2 genotypes in controls is not in the Hardy-Weinberg equilibrium as reported in Caucasian [19] and Asian [26] populations. Although the explanation is not known, the random recruitment of the healthy individuals, the reproducible genotyping method and the consistence with the Hardy-Weinberg equilibrium in several other polymorphic loci [8,9,23], suggests that the controls in the present study may reasonable be used in case-control investigations.

Our results suggest that the studied polymorphism in the promoter region of the *MMP9 *gene is associated with the risk of the development of lung cancer. Thus, individuals with the *MMP9 *-1562 T/T genotype have shown a protective effect against the development of lung cancer compared to the reference genotype (-1562 C/C). In relation to survival analysis, the *MMP2 *-735 T/T genotype was significantly associated with an unfavourable survival prognosis in patients with NSCLC.

The MMP family comprises 23 human enzymes that traditionally have long been associated with cancer invasion and metastasis because of their ability to degrade the extracellular matrix. However, recent studies have showed that the roles of MMPs in tumour development and metastasis are much more complex than was originally envisioned. In vitro and animal studies have demonstrate that MMPs are also the key mediators of growth factor activation, bioavailability and receptor signalling, cell adhesion and motility, apoptosis and survival mechanisms, angiogenesis, and inflammatory responses and immune surveillance [10]. In this sense, high levels of MMP2, MMP3 and MMP9 proteins have been implicated in several malignancies including oesophageal, renal, head and neck, oral, colorectal, NSCLC, breast carcinomas and melanomas [27-34]. However, recent studies have shown that several members of this family, including MMP9, which were originally recognised as pro-tumourigenic proteases [35], provide a protective effect in different stages of cancer progression [36,37]. These experimental analyses support the results obtained in our study where individuals with the *MMP9 *-1562 T/T genotype showed a decreased risk of developing lung cancer. Only two studies have evaluated the association between the *MMP9 *-1562 C/T polymorphism and lung cancer risk, both finding a non-statistically significant association [19,26]. Several hypotheses can explain this apparent discrepancy. First, Zhou et al. carried out their study among the Chinese population, whereas all individuals included in our study were Caucasians. In this sense, numerous differences have already been reported concerning genotype frequencies and cancer susceptibility between Asian and Caucasian populations. For example, a recent meta-analysis for the *MMP1 *1 G/2 G polymorphism found a statistically significant association with cancer risk in European populations, whereas no association was found in Asian populations [18]. Second, Rollin et al. analysed the risk in 90 cases and 90 controls, though this sample size was too small to yield a real association [19].

With regard to the *MMP3 *-1171 5A/6A polymorphism, two studies have investigated the association between this polymorphism and lung cancer risk, showing a non-statistically significant association [17,18]. Thus, our findings are consistent with previous studies.

Finally, two studies have evaluated the lung cancer risk for individuals with the -735C/T polymorphism in the *MMP2 *gene, showing an 1.6-fold increased risk associated with the -735C/C genotype in Asian populations [26] and no significant association in Caucasian populations [19], which is in line with our results (although it should be noted that Rollin's study includes only 90 cases).

Alternatively, in this study, we investigated the effects of these three polymorphisms on the survival time of 816 lung cancer cases, subdivided on the basis of their different histopathological presentation and clinical stages (NSCLC: I, II, III, IV and SCLC: extended or limited). To date, a large number of studies have investigated the relationship between variants in the *MMP2, 3 *and *9 *genes and cancer susceptibility or metastasis, including lung cancer [26,38]. However, only three studies have explored the relationship between the polymorphisms in these *MMPs *and survival rates among patients with NSCLC [19,20,22]. Rollin et al. explored the effect of the *MMP9 *-1562 C/T and the *MMP2 *-735C/T polymorphisms in NSCLC survival among Caucasian patients and found that the *MMP9 *-1562 C/T polymorphism did not present a significant increase in survival rate, in accord with our results (although it should be noted that Rollin's study includes only 90 patients). However, the homozygous individuals for the *MMP2 *-735C allele had a shorter survival time than those carrying the T allele (*P *= 0.02), and Cox's proportional hazard regression analysis demonstrated that this polymorphism was an independent risk factor for a shortened survival time (*P *= 0.045) [19]. Similarly to the risk analysis, one possible reason for this discrepancy is the relatively small sample size (90 patients). In another study, Heist et al. investigated the association of five polymorphisms, including the *MMP3 *-1171 5A/6A polymorphism, in 382 patients with stage I lung cancer, finding that individual carriers of a variant genotype did not present a significant increasebetter in survival rate. To verify these results in our study, we analysed the effects of the *MMP3 *-1171 5A/6A polymorphism in the group of patients with stage I NSCLC and obtained similar results (adjusted HR = 1.20; 95% CI: 0.72-2.00) [data not shown]. Finally, a recent study of 561 NSCLC patients in a Chinese population analysed the effects of 14 SNPs in *MMPs *genes in the overall survival of patients with NSCLC and found that the *MMP2 *-735 T/T and *MMP3 *-1171 5A/5A genotypes did not decrease survival time. However, large sample size studies in Caucasian and Asian populations are needed to corroborate and validate these findings.

Our study is the first to analyse the effects of three polymorphisms in the *MMPs *genes on the survival time in SCLC patients. Our results indicated that the *MMP2 *-735 T/T genotype did not show significant differences in survival time in patients with SCLC, whereas it was associated with a significant decrease in survival among patients with NSCLC. Studied polymorphisms in the *MMP3 and MMP9 *genes showed no effect on the survival time in both NSCLC and SCLC. The discrepancy of these findings may be explained by different expressions of MMPs between SCLC and NSCLC tissues. Thus, one study among 45 patients with SCLC, where expression was evaluated by Immunohistochemistry (IHC) for several MMPs, showed a wide expression for all MMPs, except for MMP2, whose expression was not detected [39]. However, a recent meta-analysis showed that MMP2 is highly expressed in NSCLC patients and that decreased the survival time [40]. These results seem to show that some MMPs may be more specific to NSCLC tissues than to SCLC tissues.

Our study has several strengths, including high participation of eligible cases (rate of 91.4%) and quite a large sample size from a homogeneous population of similar ancestry (879 cases and 803 controls). Furthermore, all of our cases were pathologically confirmed, and we finally applied a strong quality control for genotyping. Inevitably, the use of hospital-based controls is a potential limitation. Although there is always a chance of recall bias because information on confounding variables was obtained retrospectively, the estimations obtained for the most important confounding variable (tobacco) was nevertheless in line with the literature. Even though our sample size is quite large because of the low allelic frequency in the studied polymorphisms, our patient and control groups with variant genotypes were probably not large enough to study the impact of these gene polymorphisms on lung cancer risk. Therefore, further studies with larger populations are necessary to reach conclusions. Along the same line, the approach taken in this manuscript (over-simplified with only 1 genetic variant in each MMP gene) is a limitation. To properly assess the association between the *MMP *genes and lung cancer risk and survival, it would be preferable to take a tagging variant approach.

## Conclusions

In conclusion, our findings show a relationship between the *MMP9 *variant genotype and protection against the development of lung cancer and that the *MMP2 *variant genotype modifies the survival time in NSCLC patients. However, it is important to consider that *MMPs *polymorphisms may not occur as independent events and could be associated with other polymorphisms in the genome. Thus, there is still an important role for these studies in candidate genes even in the current GWAS era.

## Competing interests

The authors declare that they have no competing interests.

## Authors' contributions

PGA carried out molecular genetic studies and drafted the manuscript. TP participated in the patient enrolment. AGA and AFS performed the statistical analysis. MFLC participated in the molecular genetic studies and revised the manuscript. AT conceived the study, participated in its design and coordination, and revised the manuscript. All authors read and approved the final manuscript.

## Pre-publication history

The pre-publication history for this paper can be accessed here:

http://www.biomedcentral.com/1471-2407/12/121/prepub

## Supplementary Material

Additional file 1 **List of pathologies accepted for controls**. Click here for file
