# Supplementary material for: Genetic polymorphisms in MMP 2, 9 and 3 genes modify lung cancer risk and survival
Source: BMC Cancer. 2012 Mar 28;12:121. doi: 10.1186/1471-2407-12-121 (PMC3350430; doi:10.1186/1471-2407-12-121)
Supplement: Additional file 1 — List of pathologies accepted for controls. [file 1471-2407-12-121-S1.DOC]

**Additional file 1**

**List of pathologies accepted for controls**

1. Diseases of the nervous system and sense organs

1.a. Cataract (ICD-366)

2. Diseases of the circulatory system

2.a. [Varicose veins](http://en.wikipedia.org/wiki/Varicose_veins) of [lower extremities](http://en.wikipedia.org/wiki/Lower_extremities) (ICD-454)

2.b. [Hemorrhoids](http://en.wikipedia.org/wiki/Hemorrhoids) (ICD-455)

2.c. Varicose veins of other sites

2.c.1. Scrotal varices (Varicocele) (ICD-456.4)

3. Diseases of the digestive system

3.a. Diseases of oral cavity, salivary glands, and jaws

523.0 Acute gingivitis

523.2 Gingival recession

523.3 Aggressive and acute periodontitis

523.5 Periodontosis

523.6 Accretions on teeth

523.9 Unspecified gingival and periodontal disease

3.b. Appendicitis (ICD-540-543)

3.c. Hernia of abdominal cavity (ICD-550-553)

3.d. Other diseases of intestines and peritoneum

3.d.1. Intestinal obstruction without mention of hernia (ICD-560)

3.d.2. Diverticula of intestine (ICD-562)

3.d.3. Other disorders of intestine (ICD-569)

3.e. Other diseases of digestive system

3.e.1. Cholelithiasis (ICD-574)

4. Diseases of the genitourinary system

4.a. Diseases of male genital organs

4.a.1. Hydrocele (ICD-603)

603.0 Encysted hydrocele

603.8 Other specified types of hydrocele

603.9 Hydrocele, unspecified

5. Diseases of the skin and subcutaneous tissue (ICD-680-709, except 692, 693, 694, 696, 707)

6. Diseases of the musculoskeletal system and connective tissue

6.a. Osteopathies, chondropathies, and acquired musculoskeletal deformities

6.a.1. Acquired deformities of toe (ICD-735)

7. Injury and poisoning

7.a. Fractures (ICD-800-829)

7.b. Dislocation (ICD-830-839)

7.c. Sprains and strains of joints and adjacent muscles (ICD-840-848)

7.d. Intracranial injury, excluding those with skull fracture

7.d.1. Concussion (ICD-850)

7.e. Internal injury of thorax, abdomen, and pelvis (ICD-860-869)

7.f. Open wounds

7.f.1. Open wound of upper limb (ICD-880-887)

7.f.2. Open wound of lower limb (ICD-890-897)

7.g. Injuries to blood vessels (ICD-900-904)

7.h. Superficial injury of face, neck, and scalp except eye (ICD-910)

7.i. Superficial injury of trunk (ICD-911)

7.j. Superficial injury of shoulder and upper arm (ICD-912)

7.k. Superficial injury of elbow, forearm, and wrist (ICD-913)

7.l. Superficial injury of hand(s) except finger(s) alone (ICD-914)

7.m. Superficial injury of finger(s) (ICD-915)

7.n. Superficial injury of hip, thigh, leg, and ankle (ICD-916)

7.o. Superficial injury of foot and toe(s) (ICD-917)

7.p. Superficial injury of eye and adnexa (ICD-918)

7.q. Superficial injury of other, multiple, and unspecified sites (ICD-919)

7.r. Contusion with intact skin surface (ICD-920-924)

7.s. Crushing injury (ICD-925-929)

7.t. Other and unspecified effects of external causes (ICD-990-995)
